# Policy challenges for the pediatric rheumatology workforce: Part II. Health care system delivery and workforce supply

**DOI:** 10.1186/1546-0096-9-23

**Published:** 2011-08-15

**Authors:** Michael Henrickson

**Affiliations:** 1 Division of Rheumatology, MLC 4010, Cincinnati Children's Hospital Medical Center, 3333 Burnet Avenue, Cincinnati, OH, 45229-3039, USA

**Keywords:** pediatric rheumatology, pediatric subspecialty, policy, workforce

## Abstract

The United States pediatric population with chronic health conditions is expanding. Currently, this demographic comprises 12-18% of the American child and youth population. Affected children often receive fragmented, uncoordinated care. Overall, the American health care delivery system produces modest outcomes for this population. Poor, uninsured and minority children may be at increased risk for inferior coordination of services. Further, the United States health care delivery system is primarily organized for the diagnosis and treatment of acute conditions. For pediatric patients with chronic health conditions, the typical acute problem-oriented visit actually serves as a barrier to care. The biomedical model of patient education prevails, characterized by unilateral transfer of medical information. However, the evidence basis for improvement in disease outcomes supports the use of the chronic care model, initially proposed by Dr. Edward Wagner. Six inter-related elements distinguish the success of the chronic care model, which include self-management support and care coordination by a prepared, proactive team.

United States health care lacks a coherent policy direction for the management of high cost chronic conditions, including rheumatic diseases. A fundamental restructure of United States health care delivery must urgently occur which places the patient at the center of care. For the pediatric rheumatology workforce, reimbursement policies and the actions of health plans and insurers are consistent barriers to chronic disease improvement. United States reimbursement policy and overall fragmentation of health care services pose specific challenges for widespread implementation of the chronic care model. Team-based multidisciplinary care, care coordination and self-management are integral to improve outcomes.

Pediatric rheumatology demand in the United States far exceeds available workforce supply. This article reviews the career choice decision-making process at each medical trainee level to determine best recruitment strategies. Educational debt is an unexpectedly minor determinant for pediatric residents and subspecialty fellows. A two-year fellowship training option may retain the mandatory scholarship component and attract an increasing number of candidate trainees. Diversity, work-life balance, scheduling flexibility to accommodate part-time employment, and reform of conditions for academic promotion all need to be addressed to ensure future growth of the pediatric rheumatology workforce.

## Background

For children with rheumatic conditions, the available pediatric rheumatology (PR) workforce in any country mitigates their access to care. While the subspecialty experiences steady growth, a critical workforce shortage constrains access. A central mission of the PR workforce is to provide children with access to care and superior clinical outcomes. Part I detailed the unique pattern of challenges facing the PR workforce resulting from obsolete, limited or unavailable exposure to PR. Acting synergistically, the first barrier comprises three challenges. These are: a) absent or inadequate recognition or awareness of rheumatic disease by primary care providers, patients and their families; b) referral patterns that commonly foster delays in timely diagnosis; and c) primary care providers' inappropriate or outdated perception of outcomes. The second major barrier facing the PR workforce is the combined adverse effect of market competition, inadequate reimbursement and uneven institutional support. This barrier fosters a proliferation of varying models of PR care delivery. Generally, these versions of care delivery do not effectively improve clinical outcomes in a reliable, planned manner of longitudinal care.

Part II of this three-part review explores two additional national barriers and potential policy solutions for the United States (US) PR workforce. The third and fourth barriers are: 3) compromised quality of care due to current health system delivery, with limited patient access to self-management programs and multidisciplinary team care; and 4) an insufficient workforce supply available to meet the current demand.

## Barrier 3: Compromised Quality of Care Due to Current Health System Delivery, with Limited Patient Access to Self-Management Programs and Multidisciplinary Team Care

The Institute of Medicine identified health care quality deficiency in its landmark report *Crossing the Quality Chasm*. This report attributed the gap in quality to a fundamental problem in health system delivery design. The health care of adults with chronic illness continues to be a major health policy issue because of the mediocre quality of care [1]. In the US, approximately 40% of patients do not receive adequate health care once a chronic condition becomes apparent [2]. Of the care provided, 20% is clinically inappropriate [3]. Deficits in clinical quality and rising patient, provider and policymaker dissatisfaction reflect the mismatch between enduring needs of patients with chronic health conditions and a health care delivery system principally organized for the diagnosis and treatment of acute conditions [4-6]. Quality has further suffered due to a combination of the system's inability to meet demands for medical care due to both poor organization of health care delivery and constrained access to information technology. Rapid increases in chronic disease prevalence and technological complexity stemming from scientific advances have created these escalating demands. Since system design is the focus of needed change, trying harder with the current system will not achieve improvements [2]. The desire for an easy solution to rising costs and poor quality in US health care has partially limited understanding the vital role of care redesign in improving health outcomes [4]. Quality improvement must generate evidence regarding system redesign that produces better care and methods to achieve such transformation than is currently practiced [7].

Chronic health conditions essentially present a common set of challenges to patients and their families. All involve coping with symptoms, disability, emotional effects, demanding lifestyle changes, and the need to obtain effective medical care, often accompanied by complex treatment regimens. Care received frequently fails to deliver best clinical practices. In the biomedical model, providers possess the knowledge and they are accountable for the patient's health. Patient education occurs unilaterally. Current care complexity frequently leaves patients unable to know how to perform self-care after they leave the clinic. Poorly coordinated care, especially for patients with chronic health conditions, often leads to medical errors, higher costs and unnecessary pain [8].

**Figure 1:**
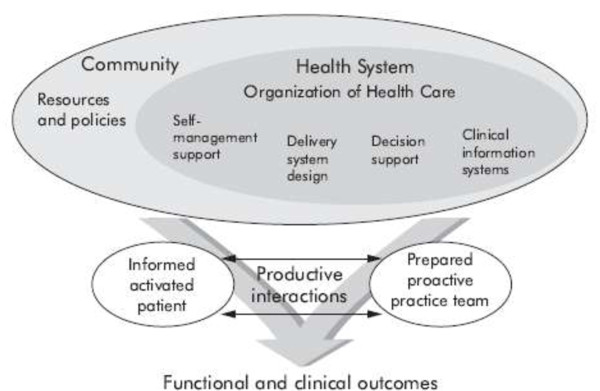
**Wagner's Chronic Care Model **[3].

Wagner developed the chronic care model (CCM), depicted in Figure 1, based on clinical experience and medical evidence to promote improvements in the care of patients with chronic illnesses [9]. Its aim is to transform care delivery from acute and reactive to anticipatory and population-based. Specifically, six inter-related system changes of the CCM intend to achieve patient-centered, evidence-based care. These changes involve a combination of effective team care and planned interactions; self-management support; strengthened, effective use of community resources; integrated decision support; patient registries; and supportive information technology [10]. The CCM incorporates productive interactions between organized, proactive practice teams and well-informed motivated patients. Standards of care and treatment aims are clear and evidence-based. Care management links to a patient registry which collects data, schedules care, creates reminders, and supplies data to providers regarding patients' attainment of quality indicators. Self-management education, shared goal-setting, links to community organizations, and written care plans support patients and families [9]. Effective teams experiment with tests of change affecting system improvement. In turn, systematic changes affect care processes for individual patients and ultimately for patient outcomes.

US reimbursement policy (outlined in Part I, Barrier 2) and overall fragmentation of health care services pose particular challenges for widespread implementation of the CCM. There is also a lack of coherent policy direction for the management of high cost chronic diseases in US health care [3]. Musculoskeletal disorders rank third among the most costly diseases; 78% of their total cost is due to indirect costs [11]. Recommended services and modes of delivery of the CCM are poorly reimbursed or non-reimbursed in most fee-for-service plans. The combined effects of unsupportive reimbursement, a doubtful business case, and the additional effort required by busy practices all limit comprehensive fulfillment of the CCM, except by very large networks or institutions. Reimbursement policies and the actions of health plans and insurers are the most consistent barrier to chronic disease improvement [7]. Current fee-for-service reimbursement policies, especially involving the Centers for Medicare and Medicaid Services, are most problematic for the provision of non-visit methods of care interaction, self-management support and group interactions. Such policies persist despite very substantial evidence of the efficacy and efficiency of these care delivery methods. Extensive adoption of the CCM in the US will require broad-based political, financial and community support.

Self-management programs are designed to improve the self-care of individuals with chronic health conditions. Established programs are multi-component and integrate information about chronic disease. Additional features of self-management include an overview of its principles, motivational interviewing techniques, cognitive symptom management, coping strategies for negative feelings, behavioral contracts with action planning, and effective communication with family and clinical providers. The self-management intervention aims to develop a patient's perceived capacity to control a number of disease features. This is accomplished by improving self-efficacy through skill mastery, modeling, persuasive communication and reinterpretation of symptoms [12].

Children and youth with chronic health conditions are particularly vulnerable to fragmented, uncoordinated care [13]. This demographic comprises 12-18% of the US pediatric population [14,15]. These children typically have far more unmet needs related to important medical services than do the majority of children [16]. Consequently, children with special health care needs likely receive less than best possible care. Poor, uninsured and minority children may be at a heightened risk for inferior coordination of services [17,18].

The PR workforce strives to provide chronic rheumatic disease care in a delivery system characterized by these numerous barriers to effective longitudinal treatment. Self-management is uncommonly employed in PR practices. As an element of the CCM, the multidisciplinary team's role is well-established in improving outcomes for patients with chronic health conditions. In rheumatic diseases, one observational study and another randomized controlled trial in adult patients with rheumatoid arthritis involved multidisciplinary care teams. Both studies indicated treatment effectiveness for the team approach [19,20]. A proof-of-concept, randomized controlled trial in adult patients with systemic sclerosis indicated effective treatment for several outcome measures involving overall physical health compared to regular outpatient care [21].

Nevertheless, many PR clinicians remain either unconvinced of the need for a team approach to chronic disease care, or their work setting is unsupportive. The stated primary rationale for this lack of support is typically financial. In turn, this underscores currently unsupportive, fee-for-service reimbursement policy for chronic disease care providers. The impact remains greatest for the children with chronic conditions. In the US health care delivery system, health outcomes and quality of care unfortunately remain mediocre.

## Solution 3: Implementation and Extension of the Chronic Care Model

Modern chronic illness care requires productive interactions between the practice team, patient and her/his family. Along with clinical care, such interactions must involve behavioral strategies which empower patients and families to become confident, effective and skilled in self-managing their chronic diseases. The typical acute problem-oriented visit actually serves as a barrier to care [7]. Instead, high-quality chronic disease care consistently provides the quality measures, self-management guidance, evidence-based clinical practice standards and follow-up associated with best outcomes. Interventions using self-management strategies for chronic illness indicate efficacy for the functional health domains of general health, physical function, bodily pain and mental health. Some studies have shown that the self-management program strategy alone is highly cost-effective in individuals with arthritis [12]. In addition to its economic advantage, self-management has great potential when combined with other system changes to improve outcomes.

For thousands of patients with chronic illness, time-intensive collaborative interventions applying the CCM within health care systems have established persuasive evidence, including a Cochrane Collaboration review, for process quality improvement and enhanced outcomes [9,22,23]. Coleman, et al conducted a systematic review of chronic care intervention evaluation studies. This review included only those studies containing all six elements of the CCM. Of the 56 identified studies meeting inclusion criteria, nearly 95% reported significant improvement in at least one measured outcome. Practice changes involving an increase in providers' skills and expertise, registry-based information, patient self-management support and education, and team-centered and planned care delivery led to the greatest improvements in health outcomes [10].

Fundamentally, the CCM is not a separate, immediately reproducible intervention. Rather, it is a structure within which organizations delivering care can translate general ideas for change into specific, often locally distinctive applications [10]. The CCM is "a synthesis of the best available evidence, intended to be flexible and subject to change when new evidence emerges [7]."

Fee-for-service reimbursement for pediatric chronic condition care is a hindrance to tests of change regarding personnel, visit organization or follow-up. However, small, adult medicine practices that primarily generate revenue from fee-for-service payments can achieve comprehensive system changes and demonstrate improvements in care [7]. Evidence shows that small systems perform as well as large ones in adult medicine.

Face-to-face visits are not an inevitable requirement of chronic illness care. Sufficient evidence confirms the effectiveness of using the computer or telephone for this purpose [24]. The Institute of Medicine's report encouraged increasing methods of interaction other than face-to-face visits [25]. For example, telephone contact allows for intensive, cost-effective follow-up of chronically ill patients [7]. In a variety of chronic diseases, improved outcomes associate with telephone communication [24]. Generally, current policy for fee-for-service reimbursement is not to pay for alternative methods of care coordination.

Delivery system design evidence supports multidisciplinary care visits (a.k.a., "one-stop shopping"). This design promotes access to medical and allied health services [7]. Team-based care facilitates care coordination, which reciprocally supports the health care team. Effective care coordination is best provided in the context of a real or virtual team [26]. Antonelli, et al define pediatric care coordination as "a patient- and family-centered, assessment-driven, team-based activity designed to meet the needs of children and youth while enhancing the caregiving capabilities of families. Care coordination addresses interrelated medical, social, developmental, behavioral, educational, and financial needs in order to achieve optimal health and wellness outcomes [27]."

Excellent pediatric care coordination supports and relies on team care. It reliably provides patient/family education to develop self-management skills, and plans for the transition from pediatric to adult systems of care. Among several best practice features, this level of pediatric care coordination 1) supports planned longitudinal care, 2) confers skills to families to navigate a complex health care system, and 3) ensures effective communication and collaboration along the continuum of care. These competencies need to be held individually or collectively by all clinicians, nurses, social workers, and allied health care professionals engaged as a team to support families. Such competencies should also continue to non-health professionals providing care coordination services. Under certain circumstances, subspecialty providers may serve as a medical home [27].

The monumental challenge facing care coordination is the urgent need to place the patient at the center of care, which represents a fundamental restructure of US health care. For care coordination to be done properly, necessary changes in care delivery and financing must occur. This effort's scope exceeds quality improvement work since it surpasses the boundary of a single institution or organization [8]. To realize and extend improved quality of care and best outcomes, the PR workforce will need to embrace and integrate the chronic care model into practice delivery. Specifically, self-management support should be developed in PR practices nationwide. As well, multidisciplinary team care should be established at PR practices as an evidence-based means of achieving effective care coordination. Self-management and care coordination by a proactive, prepared team are essential elements of modern health care system delivery for pediatric patients with chronic conditions. A final barrier which must be overcome to achieve this new care delivery system is the insufficient supply of available PR workforce to meet the current clinical need.

## Barrier 4: Insufficient Workforce Supply to Meet Demand

### Current Workforce Projections

In 2010, the average age of board-certified PRs was 52.2 years, with 91.2% (239) of the 262 diplomates ranging from 31 to 65 years of age (8 did not indicate their age) [28]. In 2004, although 92% of PRs treated patients, only 77% spent over 90% of their time caring for children; 32% planned to decrease their time in clinical care over the subsequent five years by one third, primarily to work in research [29]. After eliminating 28 board-certified PRs who are practicing permanently abroad, retired, employed full time by the pharmaceutical industry or are not practicing PR, and deducting 10% in the projected clinician workforce (0.33 × 0.32 = 0.1), the remainder decreases to 198, of which 186 PRs are full time clinicians (Table 1). The Health Resources and Services Administration recommends an urgent 30% increase in the US PR workforce [29].

**Table 1:** Projected US Pediatric Rheumatology (PR) Workforce Trends

	% Workforce	Number of PRs	PRs/million children
Total board-certified PRs in the US*	100%	242	3.3
PRs providing any clinical care^†^	92%	223	3.0
PRs who spend >90% of their time seeing patients	77%	186	2.5
Projected PRs after clinical work decline^§^	82%	198	2.7

Projections are based on a US population of 74 million children.

*270 ABP-certified PRs - (13 PRs practicing abroad + 6 retired + 5 employed in the pharmaceutical industry + 4 not practicing PR) = 270 - 28 = 242 US PRs.

^†^US PRs engaged in full time research = 20. 242 - 20 = 222 US PRs.

^§^One third reduction in clinical work by 32% of all PRs = (0.33 × 0.32) = overall 10% reduction.

(92% -10%) = 82% remaining workforce.

**Figure 2:**
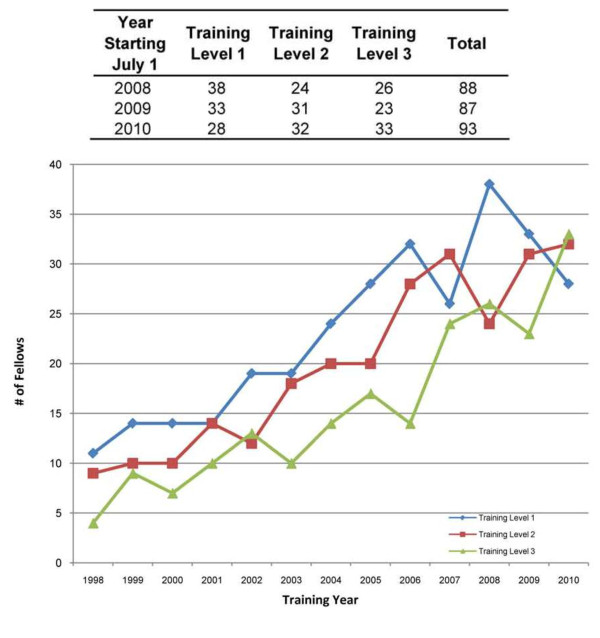
**Growth Trends for Pediatric Rheumatology Fellowship Trainees (1998-2011), with Recent Training Level Data (2008-10) **[28].

**Figure 3:**
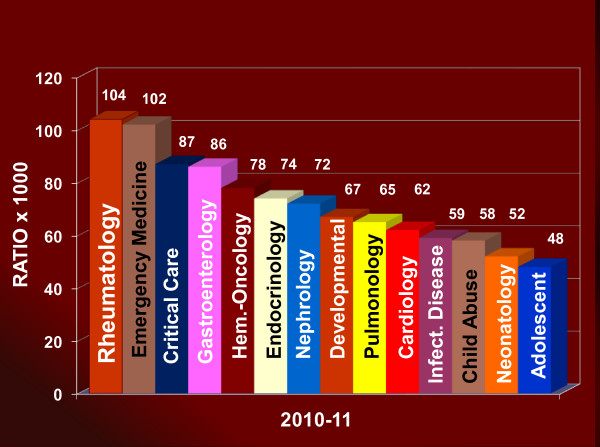
**2010-11 Comparison of 1^st ^Year Fellows/Total ABP Subspecialty Diplomates **[28].

**Figure 4:**
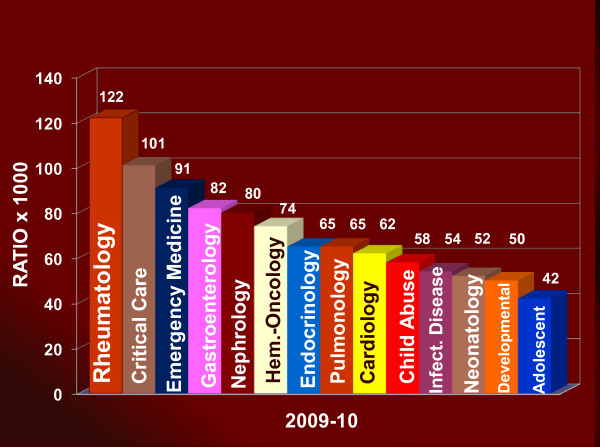
**2009-10 Comparison of 1^st ^Year Fellows/Total ABP Subspecialty Diplomates **[28]. The ratio in Figures 3 and 4 depicts the total number of 1^st ^Year Fellows by pediatric subspecialty (numerator) to the total number of ABP diplomats in the corresponding pediatric subspecialty. The ratio serves as a subspecialty comparison of proportional fellowship recruitment.

In the US, there are 32 PR fellowship training programs with a current 3-year average of 89 fellows in training (Figure 2) [28]; Canada has 3 PR training programs. International medical graduates (IMGs) comprise 31% of PR fellows at US programs; 80% of IMGs will practice in the US after completing fellowship. Despite the small number of PR American Board of Pediatrics (ABP) diplomates, the proportion of first year PR fellows to diplomates remains the highest of any subspecialty (2010-11 data shown in Figure 3, and data in Figure 4; prior data not shown) [28].

### Current Workforce Demand

**Figure 5:**
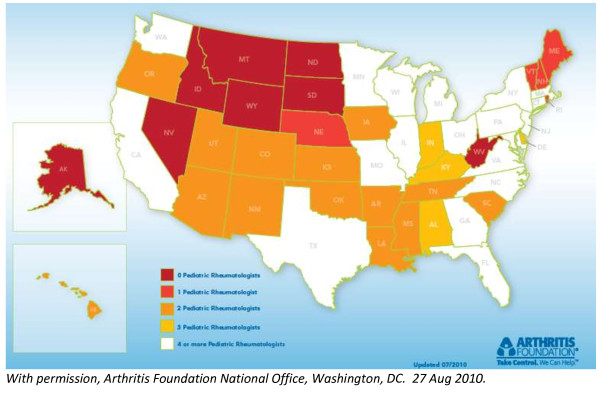
**State Distribution by the number of Board Eligible or Board Certified Pediatric Rheumatologists, 2010**.

In 2005, PR demand exceeded supply by 25-50% [30]. Currently, eight states (16%) have no PR practicing within state (Figure 5); seven are west of the Mississippi River. In 1996, 36% of U.S. medical schools had no PR faculty (45 of 125), including 42% of the 40 "primary care schools". These latter schools graduate the highest percentage of students entering primary care (range 28-44%) [31]. In 1986, 63% of medical schools lacked PR faculty, indicating the substantial progress made in the subsequent decade [32]. Thereafter, progress stalled. During the last survey of PRs at US medical schools in 2004, ~30% of schools had one PR and 22% had two PRs [33]. This staffing realistically permits clinical coverage and limited teaching; research contributions are primarily collaborative. None of the 19 US osteopathic schools have a PR on faculty. The national distribution of PRs reveals the current vulnerability of 19 states (38%) which have only one or two providers (Figure 5).

The need for PR clinical care is remarkable. The average wait time for 65% of patients is over 2 weeks [29]. Although 50% of the population below the age 18 years lives 50 or more miles from a PR, more than half live 100 or more miles from a PR. This distance is related to internist rheumatologists' involvement in the care of children, controlling for a variety of other factors [34,35]. The mean distance patients travel is 60 miles. Forty percent of patients must travel over 40 miles to receive PR care; 24% must travel over 80 miles [34]. The access to care problem is a consequence of several influences on trainees' career choice. An understanding of these influences can shape potential solutions for the chronic PR workforce shortage.

### Workforce Supply

Historically, the zenith of federal funding for graduate medical education (GME) in primary care specialties occurred in the mid-1990s. The preceding decade-long national effort to increase the number of generalist physicians succeeded. However, by the late-1990s, many pediatric subspecialties reported shortages [36,37]. The number of graduates of US pediatric residency programs that chose fellowship training fell from 33% in 1990 to 23% in 2000 [38]. Yet, these subspecialty shortages could not be attributed solely to the increased number of generalists. Additional factors contributed, including increased demand for subspecialty services, inadequate reimbursement, reduced access to subspecialists due to gate-keeping mechanisms, rising competition among subspecialists, and restrictions on the number of specialist physicians imposed by national workforce policy appropriations [39]. Subsequently, there was a 20% increase in the proportion of pediatric residents planning to pursue fellowship training (27% in 2002 to 47% in 2007). Concurrently, pediatric residents electing a generalist career decreased by 22% (62% in 2002 to 40% in 2007). Planned generalists included more women and American medical graduates (AMGs) than men and IMGs [40]. Further distinctions exist between trainees choosing subspecialty *vs*. generalist careers. Because factors associated with career choice and decision-making are at least partially attributable to the stage of one's career, it is useful to consider which factors have the greatest impact by level of training [41].

### Educational Debt and Lifestyle

Educational debt has a variable effect on career planning for pediatrics. Among medical students, debt load is a determinant of career choice. Each specialty has a specific earning potential that a student may regard as a return on investment. Students also view lifestyle, including the ability to maintain control over one's work hours, as an equally important factor [42-47].

A given specialty's residency fill rates linearly correlate with that specialty's expected income [48,49]. Although medical student debt is often cited as a deterrent from primary care specialty choice, gross income and lifetime earning potential may be more influential than educational debt [50]. Indeed, most studies do not show a linear association between debt and primary care specialty choice. Some studies indicate that students who choose primary care are more likely to have some amount of debt than those choosing other specialties. Several studies suggest that medical students choosing primary care residencies are likely to have altruistic personal values, a commitment to service, and/or "social compassion or consciousness" [51-55].

For pediatric residents, educational debt also appears to be shifting away from an influential role. Resident workforce studies initially suggested that educational debt, along with lifestyle issues, contributed to the difficulty in attracting residents to fellowships. This trend mirrored an average 22% rise in college, medical school or spouse/partner educational debt during 1997-2002 [43,56-61]. In contrast, an 8-year survey (1995-2002) at the Children's Hospital of Philadelphia indicated there was no significant association between career choice and debt of less or more than $50,000 [62]. A 2007 cross-sectional survey of 7,882 participating pediatric residents from all residency programs in the US and Canada provided similar findings. The 2007 survey was part of the Residency Review and Redesign in Pediatrics (R^3^P), an ABP project. No more than 3% of residents reported financial considerations to be the most important factor in choosing their post-residency career [63].

Since pediatric residents and young general pediatricians seek work-life balance, lifestyle issues direct their career choice [39,45,57]. Residents planning to be general pediatricians valued lifestyle and structured hours as the most important aspect of their career decision. This is especially notable for women, AMGs and third-year residents. Those planning to pursue fellowship training identified a specific disease or patient population as the most important factor. This is especially remarkable for men, IMGs and residents at large centers (> 60 residents) [40]. In a 2002 survey, 90% of pediatric residents of both genders (89% of men, 91% of women) identified family considerations as the most important factor in making their employment decisions [57].

Among surveyed pediatric subspecialty fellows, only 20% rate earning potential and only 1% cite loan repayment as factors in their career pathway decision [41]. By specific subspecialty, current fellows training in higher-compensation disciplines (i.e., cardiology, critical care, and neonatology) prioritize earning potential in comparison with lower-compensated subspecialties. As for pediatric residents, such lower-earning subspecialty fellows value lifestyle as an important factor in their career choice. Conventional wisdom holds that physicians commonly gravitate to high compensation career tracks. However, economic reward appears to be only of value specifically for fellows training in these higher-compensation subspecialties. Merely 7% of practicing general pediatricians report not pursuing fellowship training due to financial issues [41].

It is possible that fellows who choose lower-compensated pediatric subspecialties represent a self-selection bias, selecting pediatrics residency for their primary care training. Overall, general pediatrics is one of the lower-compensated specialties based on hourly wages. However, general pediatrics ranks first for mean hourly wage among the three major primary care specialties (family medicine is second; internal medicine is third) [64]. A limitation to analysis and policy development that addresses the continuum of trainee decision-making is whether the available data from medical student, primary care, and pediatric subspecialty fellowship surveys can be generalized to the subspecialty of PR. This needs to be validated in future studies focused specifically to trainees planning to pursue PR fellowship and studies of PR fellows.

Public policy must confront the perception that additional financial compensation during fellowship training or in a lower-compensated subspecialty practice, e.g., PR, will attract significant numbers to specific pediatric subspecialties. Senator Tom Coburn (R-OK), a family practice physician, established a procedural practice of placing holds on proposed legislation (e.g., the Arthritis Prevention, Control, and Cure Act) in his Senate Health, Education, Labor and Pension Committee role. Senator Coburn has carefully built a reputation for fiscal conservatism and marked restrictions on federal spending. He stated at an October 2008 Town Hall meeting in Oklahoma City that "the way to create more PRs is to pay them more" [65]. This is the certainty held by a number of legislators in pivotal Congressional positions who have the capacity to advance subspecialty health care workforce legislation. Contrary to this dogma, the greatest motivation cited by pediatric subspecialty fellows in choosing their career is an interest in a specific disease or patient population [40]. PRs need to meet such overt, public displays of "market forces" misinformation directly.

With enactment of two new, major US health care bills, Public Law (PL) 111-152 Reconciliation Act of 2010 and PL 111-148 Patient Protection and Affordable Care Act, policy can now focus on pediatric specialty shortage areas and under-served populations [66,67]. PL 111-148 specifically promotes financial incentives through loan repayment (provides $35,000 for each year of service, with a 3-year loan limit) to foster recruitment to lower-compensated subspecialty careers.

Unfortunately, the Affordable Care Act is an authorization law, not an appropriations law. Opposition to the Act remains fierce, manifesting as a two-prong strategy. The first prong is a federal court appeals process based on the premise of the law having an unconstitutional basis. This is chiefly founded on the requirement for citizens to purchase health insurance or be fined for non-compliance. The second prong involves numerous efforts to strip funding from its specific programs, effectively neutralizing the law from being applied.

In this manner, The US House of Representatives passed bill 1217 in April 2011. This bill would convert the Affordable Care Act's Prevention and Public Health Fund from mandatory to discretionary status. The purpose of the Prevention and Public Health Fund is to supply a dedicated stream of resources to pay for much-needed preventive services and public health. This Fund specifically includes support to states and communities across the country committed to strengthening the pediatric and primary care workforce. Loss of the mandatory funding requires Congress to set aside appropriations to support the Fund in the federal budget process each year. Annual funding is highly unlikely in the current economic state of massive federal debt, Congressional budget battles, and political expediency of discretionary spending reductions. This example illustrates how challenging it is to obtain workforce support through the federal authorization and appropriations process. It also typifies the fragile state of federal financing commitment to pediatric subspecialty training.

Table 2 outlines lifestyle factors identified by pediatric generalists and subspecialists considered most important by gender and medical graduate source [41,62,68,69]. The Affordable Care Act limits loan applicants to US citizens or permanent legal US residents. However, currently underserved areas may not predominantly attract AMGs. As a group, AMGs do not highly value underserved geographic locations as a career lifestyle factor. Underserved areas demand deferral of lifestyle features, e.g., family considerations and ability to control work hours. Instead, AMGs favor job security, a specific disease and a patient population of interest. Alternatively, IMGs place value on acceptable income without a stated priority on geographic location, family considerations or ability to control work hours. This different value hierarchy from AMGs may incline IMGs to serve as the initial providers in underserved regions (e.g., states with no in-state PR). Future observational studies will be important to help project the demographics of PRs and other pediatric specialists who establish practices in underserved geographic regions.

**Table 2:** Lifestyle Factors Valued Most by Pediatric Generalists *vs*. Subspecialists [41,62,68,69]

Lifestyle Factor	Generalists	Subspecialists
Family considerations	Yes for both genders	No
Geographic location	Yes for women, AMGs	No
Future colleagues	Yes for women, AMGs	No
Control over work hours	Yes for women	No
Teaching and research opportunities	Yes for men, IMGs	Yes
Job security	Yes for IMGs	No
Acceptable income, salary	Yes for IMGs	Yes for IMGs only
Earning potential	Yes for men	Yes for men
Technical skills	No	Yes
Subject matter	No	Yes
Part-time work	Yes, especially for women	*Unknown (no data)*
Avoidance of burn-out	No	Yes

**Figure 6:**
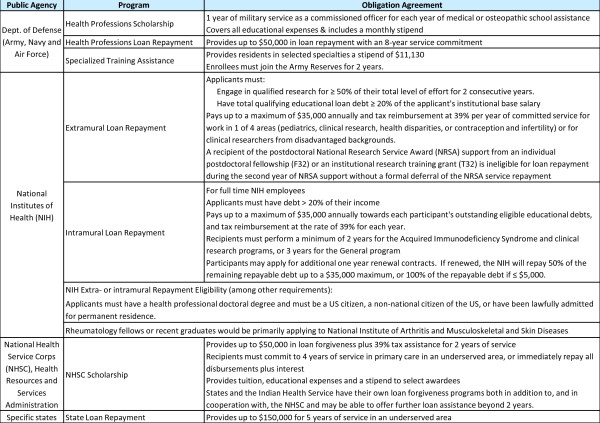
**Service Obligation Programs Available for Loan Repayment **[70,71].

Reliable income is a feature of several other service-based repayment options. These include service in the National Health Service Corps, and practice in urban or rural underserved areas. Additional service options include areas with large vulnerable racial, ethnic, or cultural patient populations. Adequate facilities, staff and competitive salary support provide incentives for interested PRs [39]. For perspective, a 2001 national survey of 935 practicing pediatric generalists and subspecialists indicated mean salaries of $125,679 for general pediatricians and $156,284 for pediatric subspecialists [68]. Figure 6 summarizes available public agency service obligation programs for loan repayment [70,71]. Focusing attention on characteristics which appeal to those who pursue a subspecialty fellowship may improve workforce recruitment. Another vital element in the career decision-making process is timing.

### Timing of Career Decision

In a 2007 survey of 755 physicians entering their second or third year of pediatric subspecialty fellowship, 43% of respondents decided to pursue subspecialty training before residency. Another 24% made this decision during their first year of residency [72]. Notably, this choice was not topic-focused for many prior to residency. Forty five percent decided on their subspecialty focus by their first year of residency. In areas other than neonatal and critical care, fellows were more likely than their peers to decide to pursue subspecialty training before residency, especially male fellows. Female fellows were inclined to decide during their second year of residency. The majority of fellows made the subspecialty training decision just before the fellowship application deadline (August of the 3^rd ^year of residency). Another survey conducted from 2002-2006 focused on 781 practicing pediatric subspecialists between 1-5 years post-residency training. The timing of their decision to pursue subspecialty training varies by their level of training. Thirty six percent decided before residency, 19% during their first year and 27% in their second year of residency [34]. Among pediatric residents, the proportion planning to pursue subspecialty fellowships did not vary remarkably during their 3-year training interval. The proportion of residents that state a specific disease or patient population as the most important factor in their career decision shows little variance throughout residency. This implies sustained focus once it occurs [40].

### Work-related Stress

In a 2001 national survey of 935 practicing pediatric generalists and subspecialists, the subspecialists reported caring for more than twice as many patients with complex medical and psychosocial problems than did general pediatricians. Subspecialists spent more than 3 times the number of hours in the hospital each day, and worked 9 hours more in total each week than generalists. It is routinely evident to trainees that pediatric subspecialists work longer hours and take more call than general pediatricians [68]. Compared with general pediatricians, pediatric subspecialists had significantly higher levels of job stress and burnout. Although not statistically significant, subspecialists had a greater tendency than general pediatricians to a) leave their current job in the subsequent 2 years and b) plan to change their specialty in the ensuing 5 years [68]. Whether this data would be reproduced in a contemporary survey is unknown. This 2001 survey occurred during the nadir of the pediatric subspecialty supply. Such issues of high job stress and risk of burnout leads to the "musical chairs" phenomenon. This effect serves to destabilize PR programs and limit growth at both nascent and established centers [73]. Policy initiatives allowing subspecialists to lighten their clinical workload, enhance their relationships with patients, and promote work-life balance must be considered [68].

## Solution #4: Fundamental Needs of the Next Generation

### Two-year Fellowship Option

A 2007 survey of 755 pediatric subspecialty fellows indicated a majority (64%) would not shorten their general pediatrics residency if they could. However, 52% would choose a 2-year fellowship over the current 3-year format [40]. Institutionalized over two decades ago, the 3-year fellowship sets pediatric subspecialty training apart from many internal medicine subspecialties [74,75]. The American Academy of Pediatrics Section of Rheumatology conducted an online survey in 2006 of its then 148 members, asking if they would support a 2-year fellowship that preserved the mandatory scholarly activity component. Of the 90 respondents (61% response rate), a solid majority (70%) supported this proposal [76]. The majority's desire to shorten fellowship training should generate a careful discussion regarding the skills necessary for a PR [40]. This is especially prompted by the existing critical workforce shortage.

The ABP's R^3^P project examined residency content and length in view of evolving pediatric practice and workforce needs [77]. The R^3^P project proposed a continuous improvement approach, i.e., a method of continuous evaluation and innovation, to system-wide change rather than specific recommendations [78]. The project promoted innovative solutions through a developing partnership with the Residency Review Committee for Pediatrics. By way of decentralization, the Initiative for Innovation in Pediatric Education (IIPE), the implementation entity for R^3^P's goals, avails training programs with a selection of strategies for change at the program level. IIPE is funded by the Federation of Pediatric Organizations [79]. These strategies capitalize on situation-specific opportunities and, ideally, supportive hospital partners. Similarly, review of PR fellowship content and duration would benefit from the continuous improvement process, seeking innovate strategies such as the 2-year training option.

The ABP set a precedent by adopting the alternate pathways listed in Table 3 for residency training to adapt to the needs of those pursuing careers in basic research [80]. ABP leadership reasoned that such candidates benefit from less clinical training and more research training than required in the standard pathway. These candidates nonetheless become board eligible as general pediatricians, equivalent in every respect to those with 50% more clinical training. The ABP recently eliminated the Special Alternative Pathway. Those pediatric residents first entering their training in 2010 were the last group eligible to petition for this pathway.

**Table 3:** Available Fellowship Alternatives Approved by the American Board of Pediatrics [80]

	Alternative Training Pathway
1	Subspecialty Fast-tracking Pathway (for those with a PhD degree or similar research accomplishment)
2	Accelerated Research Pathway (for physician scientists)
3	Integrated Research Pathway (completion of a PhD is a prerequisite)
4	Dual and Combined Subspecialty Training Pathways (this requires an Internal Medicine/Pediatrics residency)

In comparison to such alternate pathways, a policy proposing a pathway that does not shorten clinical training and does not remove any other requirements for fellowship training, specifically the requirement for scholarly activity, should be considered equally valid. The attractiveness of PR may be constrained by the assumption that training currently requires a 3-year fellowship. Pediatric residents interested in pursuing a clinically-focused track may find the additional year unappealing. Thirty-seven percent of generalists five years post-residency were more likely to choose a subspecialty if combined residency and subspecialty training were five years instead of six [81]. If this substantial proportion pursues abbreviated subspecialty training, a potential, unintended consequence of offering a 2-year fellowship would be a decrease in available general pediatricians.

Career choices of internal medicine residency graduates' demonstrated this trend in the past decade. However, AMG internal medicine residents with substantial educational debt ($100,000 - $150,000) are more likely than those without debt to pursue subspecialty training [82-84]. Since very few pediatric residents make their career choices based on educational debt, a proportionate rise in pediatric subspecialty fellows will likely be motivated by residents' academic interests.

The necessity of a 3-year fellowship for producing excellent clinicians has never been established by any rigorous scientific method. The justification has been historical precedent and consensus. Proponents assume three years is a reasonable, minimal training period for a research-oriented academic physician [85]. This is an educational policy decision that was made with the best intentions, but without substantive data on the educational outcomes. The mandate of current training periods should be established on the strength of data, not assumption [56].

The scholarship component of a 2-year fellowship will remain the most considerable programmatic hurdle. Nevertheless, this requirement could be accomplished while maintaining the requirements for ABP subspecialty certification. The ABP's Scholarship Oversight Committee determines whether a specific scholarly activity is appropriate for the current guidelines. The likely research participation would be a clinical project. This would need to begin early in the first year to bring the research endeavor to conclusion within a 2-year fellowship. In this scenario, the scholarly activity work product is consistent with the ABP's three fulfillment options. These are: 1) a progress report for a project of exceptional complexity, such as a multi-year clinical trial, 2) an in-depth manuscript describing a completed project, or 3) a thesis/dissertation written in conjunction with the pursuit of an advanced degree (e.g., MS or MPH). Other acceptable activities could be accomplished within the proposed alternative pathway 2-year fellowship experience. These include critical meta-analysis of the literature, systematic review of clinical practice, a critical analysis of public policy relevant to PR, or a curricular project with an assessment component. A biomedical research activity could not be confidently completed within this particular model, nor would such an activity be fully addressed in this interval. Programs offering either a 2 or 3-year fellowship could promote the development of training models for 1) Clinician Educators, 2) Public Policy programs in PR, and 3) Master Clinicians.

### Parent and Diversity Issues in Academia

Although universities are responding to demographic and cultural changes, academic medicine has been slower to adapt to its workforce's compelling need for schedule flexibility, including part-time workers, and diversification. The gender composition of PR is increasingly female. Women constitute over 60% of pediatric residents and 57% of PRs, including 67% under 40 years of age and 71% of fellows [28,33]. Co-parenting and the rising majority proportion of women are new realities without precedent in previous decades [32]. Policy should address the necessary national advocacy that can improve funding for young researchers and clinician educators. While raising families, this new workforce sector needs institutional support, encouragement and creative scheduling solutions to foster productivity. Fellowships need to accommodate training options and provide an early focus on work-life balance. Academic programs will need to reform promotion requirements. Parents of both genders need part-time practice options that appropriately address benefits and call responsibilities [39].

PR must also attract minority physicians to its homogenous workforce which is 95% Caucasian [33]. Academic centers must establish formal programs to provide role models and encouragement to minority pediatric residents to pursue fellowship training, since only 1% of underserved minority residents do so [57]. Of first-time applicants to the ABP's 2009 PR certification examination (n = 35), 77% anticipate a career in academic medicine. There is a sustained necessity for academia to address the evolving PR workforce's needs.

## Summary of Policy Recommendations

The recent authorization of federally-subsidized pediatric subspecialty loan repayment programs may not successfully increase the PR workforce. PR fellows' primary motivation is to pursue careers involving a specific disease or population, although trainees may self-select low-compensation specialties. Studies note that only 1% of pediatric subspecialty fellows cite loan repayment as an important factor in their career choice decision. Medical students who pursue lower-compensation pediatric subspecialties often defer a subspecialty focus decision until residency. These students are an important constituency for PR to reach through introduction to the subspecialty, e.g., via research projects. Since the majority of pediatric residents make their fellowship decision early in their final year, 3^rd ^year residents likely represent a low recruitment source. Rather, recruitment strategies should target 1^st ^& 2^nd ^year pediatric residents, as well as medical students.

Policy solutions need to maximize the efficiency of currently available resources nationwide. Additionally, legislative reform of current reimbursement policy which acknowledges the many non-reimbursed aspects of chronic care is imperative. Proposed policy solutions entail:

1. Widespread implementation of the chronic care model, including self-management support, that leads to improved health care system delivery

2. Increasing providers' motivational interviewing skills and expertise through self-management training programs

3. Practice changes that involve registry-based patient information, patient self-management support and education, patient/family-centered care, and team-based, planned care delivery

4. Increasing methods of provider-patient interaction other than face-to-face visits, e.g., telephone, e-mail, and telemedicine communication

5. Reimbursement reform which attends to these alternative methods of care coordination

6. Delivery system design which provides multidisciplinary team care during clinic visits

7. Offering a 2-year fellowship option that preserves the scholarship component, while establishing the evidence basis for the current 3-year duration requirement

8. Diversifying the workforce through emphasis on underserved minorities

9. Flexible scheduling to accommodate an increasing proportion of part time providers

10. Academic promotion requirement reform.

## Conclusions

The US health care delivery system is principally organized for the diagnosis and treatment of acute conditions. This system currently delivers mediocre quality of care to patients with chronic disease, abounding with inadequate, inappropriate and poorly coordinated care. Wagner's chronic care model (CCM) is an evidence-based means of transforming care delivery which is patient-centered. System changes involve a combination of effective team care and planned interactions; self-management support; strengthened, effective use of community resources; integrated decision support; patient registries; and supportive information technology. Tests of change produce system improvements. In turn, systematic changes affect care processes for individual patients and ultimately for patient outcomes. Prepared, proactive teams, coordinated care, the use of evidence-based clinical practice standards and self-management education are vital elements of appropriate, high quality processes of chronic care delivery. This approach offers substantial economic advantages with the potential to reduce US total health expenditures. Delivery system design evidence supports a multidisciplinary team approach of coordinating such complex care.

Fee-for-service reimbursement policy and overall fragmentation of US health care services hinder widespread implementation of the CCM. Children with chronic conditions are particularly susceptible to fragmented, uncoordinated care. For poor, uninsured and minority children, this effect amplifies. In the US, broad-based political, financial and community support will be required for extensive adoption of CCM. Persistent reimbursement disparities require legislative reform.

In the meantime, the demand for PR clinical services outstrips available workforce supply. The US needs a 30% minimum increase of its existing PR workforce to meet clinical demand [29]. Career decision studies of US medical trainees indicate specific determinants and timing. A subset of medical students makes career decisions based on educational debt. Generally, students who choose fellowship training do not decide on a topic focus during medical school, with the exception of high compensation subspecialties. While there is a potential for self-selection bias, residents pursuing low compensation pediatric subspecialties are motivated principally by a specific disease or patient population, especially male 1^st ^year and female 2^nd ^year residents. The majority of pediatric residents reach a decision about subspecialty focus early in the third year of residency. Beyond this period, third year residents likely represent a low recruitment source. Recruitment to PR may improve with acceptance of a 2-year fellowship option. The current requirement for 3-year fellowship duration is the result of consensus, not evidence basis. Diversity, work-life balance, scheduling flexibility to accommodate part-time employment, and reform of conditions for academic promotion all need to be addressed to ensure future growth of the PR workforce. There is a compelling need and institutional training program responsibility to diversify the PR workforce regarding part-time capacity and minority representations. Improvements in work-life balance and new roles involving equally shared parenting call for innovation in academic promotion requirements. Expansion of the PR workforce is a strategic imperative to alleviate the persistent problems of constrained access to care.

## Abbreviations

ABP: American Board of Pediatrics; ACR: American College of Rheumatology; AMG: American medical graduate; CCM: Chronic Care Model; IIPE: Initiative for Innovation in Pediatric Education; IMG: International medical graduate; NHSC: National Health Service Corps; NIH: National Institutes of Health; NRSA: National Research Service Award; PR: pediatric rheumatology/rheumatologist; R^3^P: Residency Review and Redesign in Pediatrics; US: United States.

## Competing interests

Dr. Henrickson is a current member of the American College of Rheumatology (ACR) Committee on Government Affairs. He has no competing financial interests to disclose. The content of this article does not reflect any official position or policy of the ACR.

## Authors' contributions

MH solely contributed all aspects of this article.
